# Multiple Strains of *Trichomonas gallinae* are Widespread in Reservoir Hosts and Environmental Resources in UK Farmland

**DOI:** 10.1111/mec.70520

**Published:** 2026-09-04

**Authors:** Rebecca C. Thomas, Jenny C. Dunn, Helen Hipperson, Antony J. Morris, Philip V. Grice, Keith C. Hamer, Simon J. Goodman

**Affiliations:** ^1^ School of Biology University of Leeds Leeds UK; ^2^ NERC Environmental Omics Facility (NEOF) School of BioSciences, Western Bank Sheffield UK; ^3^ School of Life Sciences Keele University Newcastle‐under‐Lyme UK; ^4^ RSPB Centre for Conservation Science, the Lodge Sandy UK; ^5^ Natural England Peterborough UK

**Keywords:** disease, parasite, transmission, trichomonosis

## Abstract

Understanding transmission routes of parasites and pathogens is critical for the development of control measures and mitigation of impacts on host populations. *Trichomonas gallinae* is a widespread parasite in columbids worldwide, and has emerged as a lethal disease in passerines, particularly finches. The postulated transmission route from columbids to passerines via spillover at shared resources has not yet been empirically tested. Here, we screen 363 birds (261 columbids and 102 non‐columbids) in the UK and France for the presence and strain identity of *T. gallinae*. To quantify potential environmental transmission, we also screened 51 food and water resources common in farmland, at 12 sites over a 2‐year period. We isolated *T. gallinae* parasites from 79% of columbids, 36% of non‐columbids, and 39% of environmental resources. Prevalence of the Type A strain was more than double in birds sampled at fed sites compared to those at unfed sites. Strain composition was mirrored in columbids, passerines, and shared food and water resources, providing compelling evidence that *T. gallinae*, especially the Type A strain, is transmitted via these shared resources. *T. gallinae* was isolated from high density feeding resources (where supplementary food was provided) nearly three times as often as low density resources (natural areas of seed‐providing vegetation), meaning that careful management of supplementary resources has the potential to reduce *T. gallinae* prevalence in wild bird populations. Further work needs to test practical means of achieving this, but ensuring any supplementary feeding is provided at low density is likely to be critical to reducing parasite transmission.

## 1 Introduction

The ability of a pathogen to infect multiple host species has been identified as a risk factor to disease emergence in both humans and domestic animals (Cleaveland et al. 2001; Taylor et al. 2001). Furthermore, these generalist multi‐host pathogens are common (Woolhouse et al. 2001). Understanding disease dynamics in such systems can enable protection of humans, livestock and species of conservation concern. Identifying reservoir hosts has important implications for parasite and pathogen distribution and persistence, because reservoir hosts can maintain it within a population or habitat, and have the ability to transmit the pathogen to a novel host.

Reservoir hosts may consist of multiple populations and even species, who contribute to the maintenance of the pathogen in varying degrees, although quantifying this can be challenging (Fenton et al. 2015; Haydon et al. 2002). Some reservoir hosts cannot maintain pathogen persistence without a main reservoir host; yet, they are still able to transmit the pathogen (Fenton et al. 2015). For example, in New Zealand, the main reservoir host for bovine tuberculosis ( *Mycobacterium bovis* ) is considered to be the brushtail possum (*Trichosurus vulpecula*), whereas the red deer (*Cervus* *elaphus* ) and ferrets (*Mustela* *furo* ) are spillover hosts (Nugent 2011). However, both red deer and ferrets can become reservoir hosts if their densities become exceptionally high (Nugent 2011). Spillover hosts can also transmit infection back to the reservoir host, known as spillback, which plays an important role in the maintenance of the pathogen in the overall population (Nugent 2011). This has important implications for any disease eradication plans, which will require management of infection in reservoir hosts and spillover hosts to prevent any risk of spillback (Nugent 2011).

The persistence of infection in a reservoir population can only be demonstrated with longitudinal studies (Haydon et al. 2002). The most powerful, practical and ethically considerate diagnostic tool currently available is the analysis of pathogen genetic material, recovered from the host. The comparison of fine‐scale variation between genetic strains of a pathogen has allowed the inference of inter‐specific transmission of *Giardia* and *Cryptosporidium* in human and animal hosts (Feng and Xiao 2011; Xiao and Ryan 2004). Single‐nucleotide polymorphisms (SNPs) in whole genome sequences revealed such fine‐scale differences between pathogen isolates that it provided evidence for recent transmissions of bacterial lineages of *M. bovis* (responsible for bTB) between badger and cattle hosts (Biek et al. 2012). The use of molecular techniques has provided valuable insights into multi‐host systems by allowing the rapid assessment of the role of each reservoir host in the epidemiology of a pathogen.

*Trichomonas gallinae* is a protozoan parasite that can cause the lethal disease trichomonosis in birds. Columbiformes, or columbids (doves and pigeons), are the natural hosts of *T. gallinae* (Stabler 1947). Although natural hosts can often carry parasites without showing clinical signs, outbreaks of trichomonosis have been reported in woodpigeons (*Columba* *palumbus* ) in southwestern Spain and Portugal (Höfle et al. 2004; Villanúa et al. 2006), in the Pacific Coast band‐tailed pigeon (*Patagioenas fasciata monilis*) in California (Girard et al. 2014), and the parasite is currently threatening the vulnerable Mauritian pink pigeon (*Columba mayeri*), where infection is a major mortality factor in nestlings and fledglings (Bunbury, Stidworthy, et al. 2008). *T. gallinae* infection prevalence can vary hugely between populations (Bunbury et al. 2007). The host range also includes birds of prey who become infected by feeding upon columbid species and consequently infect their nestlings (Boal et al. 1998).

More recently, trichomonosis has become an emerging infectious disease in passerines, mostly affecting finches, after an epidemic in Britain subsequently spread to Fennoscandia and then central Europe (Ganas et al. 2014; Lawson, Robinson, et al. 2011; Neimanis et al. 2010). One clonal strain, designated Type A, has been identified as responsible for the epidemics (Ganas et al. 2014; Lawson, Cunningham, et al. 2011; McBurney et al. 2015). Subsequent work revealed that this strain was also preponderant in a sample of British columbids that died or were shot between 2009 and 2012 (Chi et al. 2013). Included in reports are mentions of non‐finch passerines found dead at epidemic localities, including blue tit (*Cyanistes caeruleus*), coal tit (*Parus ater*), great tit (*Parus major*), yellowhammer (*Emberiza citrinella*), and sparrows (*Passer* spp.) from outbreak locations in Slovenia and southern Fennoscandia (Neimanis et al. 2010; Zadravec et al. 2012). Of these, only one blue tit was examined post‐mortem and had clinical signs consistent with trichomonosis (Neimanis et al. 2010). In the UK, gross necropsies conducted on a reed bunting (*Emberiza schoeniclus*), blackbird (*Turdus merula*), and house sparrow (*Passer domesticus*) revealed infection with the Type A strain (Chi et al. 2013). To our knowledge, there have been limited efforts in establishing the role passerines play as hosts in the epidemiology of *T. gallinae*.

Screening live passerine populations has so far only been carried out in Spain, Slovenia, Germany, North America and Canada. In Spain and Slovenia, no passerine birds, except one magpie in Spain, were found to carry the parasite (Martínez‐Herrero et al. 2014; Zadravec et al. 2016), and the genetic strain for the positively infected magpie is unknown (Martínez‐Herrero et al. 2014). However, a more recent study in Germany found 28% of passerine birds to be infected, with a relatively high strain diversity (Quillfeldt et al. 2018). In North America, several species of passerine (including finches and corvids) housed at a wildlife rehabilitation hospital in California were found to carry the Type A strain (Anderson et al. 2009). Live passerines were also sampled during a trichomonosis epidemic in Canada, with most being infected by the Type A strain although further genetic variation was detected in some individuals, suggesting multiple spillover events were responsible for the emerging disease (McBurney et al. 2015), rather than the single spillover event hypothesized to be responsible for the outbreak in the UK (Lawson, Cunningham, et al. 2011).

*T. gallinae* is postulated to have emerged in passerines by spillover from columbids at bird feeding stations (Forzán et al. 2010; Neimanis et al. 2010; Robinson et al. 2010), although there is limited evidence for this (Anderson et al. 2009; Stockdale et al. 2015). *T. gallinae* is sensitive to desiccation, and is considered more likely to contaminate water than food. However, a pseudocyst form may be resistant under stressful environmental conditions (Tasca and De Carli 2003): during this reversible life‐ stage, *T. gallinae* becomes spherical, having internalised the flagella, but no true cyst wall is present (Tasca and De Carli 2003). *T. gallinae* transmission has also been putatively linked to food resources, with a trichomonosis epidemic in Spanish woodpigeons linked to supplementary food provision (Villanúa et al. 2006). Similarly, UK columbids at farms providing supplementary food were more likely to be infected with *T. gallinae* than those at farms without supplementary food (Lennon et al. 2013). The potential role of food supplementation in disease transmission may be significant for all bird populations that rely on such resources (Becker et al. 2015).

To date, detection of *T. gallinae* in food or water sources has been sporadic. *T. gallinae* has been recovered from water containers, and the isolates successfully produced lesions in inoculated birds (Stabler 1947). Under laboratory conditions, *T. gallinae* survived in water for at least 120 min and on moist grain for up to 5 days in one study, and for up to 16 h in water with organic material in another study (Kocan 1969; Purple and Gerhold 2015); survival may be driven by low levels of dissolved oxygen (Purple et al. 2019). Other attempts involved sampling water sources that were seen to be used by columbids, and screening grain dropped by an infected bird, but only two out of fifteen water samples were positive for trichomonads, and no parasites were detected in the grain samples using culture and microscopy (Bunbury et al. 2007). In the UK, a farmyard grain pile and three artificial water sources tested at one site were positive for *T. gallinae* using culture followed by PCR detection of parasite DNA (Stockdale et al. 2015). *T. gallinae* was successfully isolated from only one sample of bird seed at sites of trichomonosis mortality in Canada using culture and microscopy (McBurney et al. 2015).

The provision of supplementary food is widespread both as a conservation measure to support wildlife populations and as a management tool to sustain introduced gamebirds (Broughton et al. 2020; Sánchez‐García et al. 2015), but may increase disease transmission by increasing local bird densities (Becker et al. 2015). Systematic provision of supplementary feed for wild birds is available in some agri‐environment schemes (AES). For example, in England, an option providing supplementary winter seed food for farmland birds' has been available with AES since 2012, (www.gov.uk 2024) mainly to address the winter ‘hungry gap’ experienced by British farmland birds between January and April (Siriwardena et al. 2008), and through a supplement, feeding can be extended to September in English counties where European turtle doves *Streptopelia turtur* breed (www.gov.uk 2023). In European turtle doves, a rapidly declining species of conservation concern in the UK (Hayhow et al. 2017) and across Europe (PECBMS 2019), *T. gallinae* is a common parasite which can cause mortality (Thomas et al. 2022; Stockdale et al. 2015). However, the prevalence of the parasite in other bird species is not well characterised and it is important to understand how the parasite can be transmitted within the wider bird community.

Here, we aim to identify potential reservoirs of *T. gallinae* in the UK and France, focussing on areas where turtle doves are still known to breed to provide a more complete picture of parasite distribution within wild bird communities and complement previous datasets from turtle doves in the UK (Lennon et al. 2013; Stockdale et al. 2015; Thomas et al. 2022), and columbids in West Africa, where turtle doves overwinter (Dunn et al. 2023). We sampled a range of bird species at the same sites at which turtle doves were monitored (Dunn et al. 2018; Thomas et al. 2022), also sampling multiple food and water resources at the same sites. We aim to test which species groups may act as reservoirs for the virulent Type A strain and identify the role that passerines may play in *T. gallinae* transmission and spillback to columbids. We identify the strains of *T. gallinae* infecting reservoir hosts and environmental resources, quantifying environmental reservoirs of T. *gallinae* to establish the extent of the issue and improve our understanding of epidemiological pathways. Finally, we test which environmental factors may influence the prevalence of infection in reservoir hosts and the percentage of environmental resources testing positive for the presence of *T. gallinae*.

## 2 Methods

### 2.1 Sites and Avian Sampling

Sampling sites for Columbiformes, Passeriformes, Galliformes and Gruiformes in the UK, and Columbiformes in France were the same as those at which Dunn et al. (2018) and Thomas et al. (2022) caught and screened European turtle doves. Samples were collected during May–July inclusive, in 2011–2015 (UK) and 2014 (France). Birds from all species groups were caught using a combination of mist nets and whoosh nets, at sites both with (UK, France) and without (UK) supplementary food in farmland. Two additional sites were used for the capture of UK passerines using mist nets in 2013–2015, at a garden site just outside Braintree, Essex (51°52′47.0″N, 0°33′10.5″E) set within an arable landscape and providing supplementary feeding in the form of bird feeders, and at a site near Salisbury, Wiltshire (51°04′02.3″N, 1°49′36.9″W) comprising semi‐natural scrub and grassland habitat with no local provision of supplementary feeding within 1 km. Birds were sampled as described elsewhere (Thomas et al. 2022), using a smaller swab (diameter 0.5 mm) for passerines but otherwise maintaining the same protocol. All birds were weighed using a digital scale (±0.1 g), and checked for visual clinical signs of *T. gallinae*; no birds were seen to have caseous lesions in the mouth or visible oesophageal tract, or to be noticeably underweight.

### 2.2 Resource Sampling

Shared food and water resources at 11 farmland sites in Essex, Norfolk, and Cambridgeshire, UK (detailed in Dunn et al. 2018) were sampled between May–August 2013–2015 to test for the presence of *T. gallinae*.

In 2013, a pilot study sampled a subset of bait sites to screen for *T. gallinae* in shared resources. Food resources sampled included temporary bait sites laid to catch turtle doves and other birds (e.g., Stockdale et al. 2015), which measured approximately 1.5 m × 15 cm and were baited as described in Dunn et al. (2018). These were often adapted from areas where supplementary seed was already being provided on the ground by the landowner. Bait sites were monitored for bird activity by camera traps (Bushnell Trophy Cam, Kansas, UK), which confirmed they were being used by a range of Columbiformes, Passeriformes, Galliformes and Gruiformes. The majority of bait sites (91%; *n* = 11) were sampled multiple times over the season. Gamebird feeders (*n* = 4), a poultry spoil heap (*n* = 1), cereal farm plots (*n* = 3) and bespoke seed‐sown trial plots (*n* = 3) as described by Dunn et al. (2015) were also sampled. Five water sources were sampled consisting of: upturned containers at ground level filled with rainwater (*n* = 1), standing water at 4 ft. above ground level (*n* = 1), water‐logged areas of farmland that persisted over the season (*n* = 2) and water troughs for livestock (*n* = 1). In 2013 a golf course in Cambridgeshire (52°52′N, 0°08′E) was also sampled due to local sightings of turtle doves.

In 2014, we followed a more comprehensive sampling strategy, whereby 11 bait sites, 2 gamebird feeders and one poultry spoil heap were sampled every 7–10 days over a period of 10 weeks, with some exceptions due to logistical constraints. This sampling frequency was chosen on the basis that *T. gallinae* is able to persist on moist grain for up to 5 days in laboratory conditions (Kocan 1969); therefore, sampling every 7–10 days should allow re‐infection rates of shared resources to be established. A similar area of nearby arable field edge/trial plots (*n* = 11) where no seed was provided was sampled at the same time to provide a control for the bait sites. These plots were chosen to represent a low density food source with only natural seed available, requiring birds to forage over a greater area. A standardised water source in the form of a 38 cm (L) × 24 cm (W) × 8 cm (D) plastic tray was placed by 10 of the bait sites and regularly topped up with tap water to maintain a certain water level. A control water source (*n* = 10) was also created, using an identical plastic tray with a plastic lid fixed securely to the top and sections cut out to allow environmental effects but prevent access by animals, although camera traps revealed that gamebirds were able to circumvent the lid and access the water. This tray was placed next to the experimental water tray with the water level also maintained, and they were both sampled at the same time as the bait sites and farm/seed mix trial plots, along with 5 natural water sources (permanent ponds). The location of the water trays next to the bait sites allowed usage by birds and other animals to also be recorded by the camera traps.

Sampling of resources involved moistening a sterile viscose swab with sterile saline solution or sterile water and running the swab through the entire length of the middle of the bait site (1.5 m). Pre‐moistening the swab was thought to increase the chance of the parasite adhering to it. The condition of the bait pile was recorded as dry, damp or wet, with the amount of seed left at the time of sampling as either none, some (less than half the original laid pile) or full (more than half of the original laid pile). Gamebird feeders were sampled by running the swab around the area where food is taken from the feeder. Farm plots, seed mix trial plots and a poultry spoil heap were sampled by running the swab along the same area of ground each week, covering a distance of 1.5 m. No repeat samples were collected from the same site at the same timepoint.

Water sources were sampled using a sterile disposable pipette to collect 0.2 mL of water. A deviation from the protocol occurred in 2014 whereby the water in standardised water trays was stirred briefly with the end of the disposable pipette before a water sample was taken. The condition of the water trays in terms of the amount of water present at the time of sampling was recorded as either dry, damp (reduced to damp algae/sedimentary remains), or full (enough water to form a level).

All swabs from birds and environmental samples, and water samples were inoculated into an individual InPouch TF culture kit (Biomed Diagnostics, Oregon), sealed and incubated at 37°C for 7 days to culture the parasite.

### 2.3 Parasite Detection

Parasite isolation, DNA extraction, PCR of the ITS 1/5.8S/ITS 2 ribosomal region (hereafter referred to as the ITS region) and the Fe‐hydrogenase region (hereafter referred to as the FeDH region), Sanger sequencing and Illumina sequencing were performed using the same methods as described in detail by Thomas et al. (2022). Each sample was screened twice with PCR. If the result was inconclusive, generally due to a smear instead of a clean band on the agarose gel suggesting low quality DNA, the sample was run a third time and if the infection status could still not be reliably determined, the sample was removed from analysis (see Appendix A for a summary of inconclusive samples). Sequencing techniques differed between years, with samples collected in 2013 analysed using PCR amplification of the ITS region according to Robinson et al. (2010) and sent off for Sanger sequencing whereas samples collected in 2014 were prepared for Illumina sequencing of both the ITS and FeDH regions (Thomas et al. 2022). The same approach to identifying molecular operational taxonomic units (MOTUs) as Thomas et al. (2022) was also adopted here. In short we used the degree of change (DOC) approach to distinguish between artefacts and biologically accurate sequences, based on the relative frequency of sequence variants found in a sample (Lighten et al. 2014; Thomas et al. 2022), Consequently, if a sequence was present with less than 50 reads in a sample, it was discarded as a potential artefact. This carried the risk of discarding sequences which were present in low numbers due to DNA degradation, which is likely to occur rapidly in environmental samples (Kocan 1969); however, these stringent measures increase the confidence we are only dealing with ‘true’ strains that successfully amplified in culture pouches; we show elsewhere that culture is essential to enable successful *Trichomonas* detection using PCR (Dunn et al. 2016).

The diet of the bird sampled was defined according to BTO fact sheets for UK species (BTO 2010) or, in the case of African species, Beauty of Birds fact sheets. The categories defined for purpose of this analysis are: granivorous (only seeds), herbivorous (only plant matter, including grains), insectivorous (only invertebrates) and omnivorous (plant matter and invertebrates, regardless of frequency). We distinguished between the long‐distance migratory turtle dove and resident (or short‐distance migratory) columbids. Climatic data were obtained from websites which held data from weather stations local to the sampling sites (http://www.tijou.co.uk/weather/mon201407.html, Essex and http://www.elyweather.org.uk/Data.html, Cambridge/Norfolk sites).

### 2.4 Statistical Analyses

To test for variance in frequency of DNA haplotypes between columbid populations and over different years we used AMOVA in Arlequin (Excoffier et al. 2005), which takes into account full DNA sequence while testing for population differences.

#### 2.4.1 *T. gallinae* in Columbids

To test for factors influencing the overall presence of *T. gallinae* in columbids, as well as for the presence or absence of each of the most common strains separately (Type A, Type C, GEO and Tcl‐1), we constructed a separate binomial general linear model in R v4.4.1 ‘Race for your life’ for each response variable. The presence or absence of *T. gallinae* or the relevant strain was designated as the response variable, with fixed effects of Year (a five‐level factor: 2011, 2012, 2013, 2014, 2015), Country (a four‐level factor: Burkina Faso, France, Senegal, UK) and host Diet (a three‐level factor: granivorous, herbivorous, omnivorous). We did not include nestling birds in these analyses, because nestling birds will not be independent either from each other, or from their parents. For strain‐specific models, we included all adult (free‐flying) birds in our analyses, including those negative for overall infection, and those for which sequencing could not confirm strain identity. The strain‐specific models did not converge when Species (a ten‐level factor) was included, so in order to capture some variation between species we included Diet as a proxy for Species. The overall model included Species but not Diet. We tested the significance of each term by comparing the model with and without the term; results are reported from the global model, and we interpreted a term as significantly influencing the response variable when *p* < 0.05.

#### 2.4.2 *T. gallinae* in All UK Birds

To test for factors influencing the presence of *T. gallinae* in all UK birds, as well as for the presence or absence of each of the most common strains separately (as above), we constructed a separate binomial general linear model in R with the presence or absence of either *T. gallinae*, or each strain separately, as the response variable. Fixed factors were Year (a five‐level factor: 2011, 2012, 2013, 2014, 2015), Month (a four‐level factor: May, June, July, Late (a combination of August and September due to low sample sizes in each month)), County (a two‐level factor: East Anglia (a combination of Cambridgeshire, Norfolk and Suffolk), Essex), Site type (three‐level factor: Farm (*n* = 10 sites), Garden (*n* = 2), Nature reserve (*n* = 1)), Fed (a two‐level factor determining whether supplementary food was regularly provided at the site (Yes; *n* = 6 sites), as opposed to the brief provision for catching purposes (No; *n* = 7 sites)), Family (a three‐level factor: Columbiforme, Galliforme, Passeriforme), and Diet (a four‐level factor: granivorous, herbivorous, insectivorous, omnivorous). Species was not included due to low sample sizes for many species, so to enable us to use all our data we instead included the Family and Diet terms to capture variation between species' ecology. As we were testing multiple factors for each model, we initially screened each factor against each response variable separately, only including those in the final model where *p* < 0.1. We did not simplify the final model; we interpreted a term as significantly influencing the response variable when *p* < 0.05. Only data from free‐flying (non‐nestling) birds were included in these analyses.

#### 2.4.3 *T. gallinae* in Environmental Resources

To test for factors influencing the presence of *T. gallinae* in environmental resources, we examined food and water resources separately because we considered different variables to be important for each. For food resources, we constructed two separate models due to only partial data being available for two variables. On the complete dataset, we conducted a binomial generalised linear mixed model (GLMM) with the presence or absence of *T. gallinae* as the response variable, and resource type within farm as nested random effects to control for multiple samples collected from the same resources over time. Fixed effects were year (a two‐level factor: 2013 or 2014), month (a three‐level factor: May, June or July), county (a two‐level factor as defined previously), rainfall (average daily precipitation (mm) recorded by nearest weather station; scaled in the model), temperature (average daily temperature (°C) recorded by nearest weather station; scaled in the model) and food resource type. Due to low sample sizes for some food resource types, we classified each resource type as either high (anthropogenic provisioning of seed) or low density (natural provision of seed through habitat management) and included this as a fixed factor (High density: bait pile, gamebird feeder, poultry spoil heap; Low density: farm plot, trial plot).

For our second food model on the reduced dataset, which included bait piles only, we also included two variables pertaining to the condition of the food resource: seed, which described the amount of food resource remaining at the site (a three‐level variable: full, some and none), and the condition of the bait pile in terms of moisture (a three‐level variable: dry, damp, and wet). Farm was included as a random effect to control for multiple samplings of the same bait site, and we removed resource type from the fixed effects previously tested. For both models, we tested the significance of each term by comparing the model with and without the term; results are reported from the global model, and we interpreted a term as significantly influencing the response variable when *p* < 0.05.

To test which variables were associated with the presence of *T. gallinae* in water resources, we initially conducted a univariate screening approach because we were testing seven variables on a relatively small dataset (*n* = 117). We included in the final model only those terms where *p* < 0.1 when screened against the response variable individually. We also tested for differences between resource types and farms, subsequently including resource type within farm as nested random effects to control for multiple samplings of the same water resource. Fixed effects were as for the first food resource model above, with the addition of a ‘state’ variable categorising the level of moisture in the water resource (full, moist, or dry), and a ‘type’ variable which identified the type of water resource (four categories: pond (a natural, permanent water source), opportunistic (transient water sources such as puddles, present after rainfall), water tray (an experimentally provided tray, or an artificial water source provided for gamebirds), and control water tray (an experimentally provided water tray with a lid intended to prevent access by birds)). We did not simplify the final model; we interpreted a term as significantly influencing the response variable when *p* < 0.05.

## 3 Results

We isolated *T. gallinae* parasites from 207 (79%) columbids across four countries, 37 (36%) of non‐columbids in the UK (comprising Passeriformes, Gruiformes and Galliformes), and 39% of environmental resources.

### 3.1 Prevalence in Columbids

The overall prevalence of *T. gallinae* in columbids differed between years, countries, and host species (Tables 1 and 2). While the model indicated that each of these variables significantly influenced the response variable (Table 2), pairwise differences were only apparent between species, with turtle doves (mean ± SE prevalence: 98.1% ± 0.9%) and collared doves (87.5% ± 8.6%) having a higher overall prevalence than woodpigeons (57.7% ± 6.9%), and turtle doves having a higher prevalence than stock doves (71.4% ± 7.1%).

**TABLE 1:** Prevalence summaries of *T. gallinae* infection in (a) Adult columbids sampled in the UK, France, Burkina Faso and Senegal between 2011 and 2015 and (b) Adult birds from non‐columbid species caught at the same sites in the UK between 2013 and 2015.

(a)							
Country	Species	2011	2012	2013	2014	2015	Total
Burkina Faso	African mourning dove (*Streptopelia decipiens*)	—	75% (*n* = 4)	—	—	—	75% (*n* = 4)
	Laughing dove (*Streptopelia senegalensis*)	—	69% (*n* = 49)	—	—	—	69% (*n* = 49)
	Vinaceous dove (*Streptopelia vinacea*)	—	50% (*n* = 2)	—	—	—	50% (*n* = 2)
France	Collared dove (*Streptopelia decaocto*)	—	—	100% (*n* = 1)	—	—	100% (*n* = 1)
	Woodpigeon (*Columba palumbus*)	—	—	100% (*n* = 2)	—	—	100% (*n* = 2)
Senegal	Black‐billed wood dove (*Turtur abyssinicus*)	—	—	—	100% (*n* = 14)	100% (*n* = 1)	100% (*n* = 15)
	Laughing dove (*Streptopelia senegalensis*)	—	—	—	100% (*n* = 30)	100% (*n* = 4)	100% (*n* = 34)
	Namaqua dove (*Oena capensis*)	—	—	—	100% (*n* = 33)	100% (*n* = 10)	100% (*n* = 43)
	Vinaceous dove (*Streptopelia vinacea*)	—	—	—	100% (*n* = 1)	100% (*n* = 1)	100% (*n* = 2)
UK	Collared dove (*Streptopelia decaocto*)	86% (*n* = 7)	100% (*n* = 1)	100% (*n* = 2)	80% (*n* = 5)	—	87% (*n* = 15)
	Feral pigeon (*Columba livia domestica*)	—	—	75% (*n* = 4)	—	—	75% (*n* = 4)
	Stock dove (*Columba oenas*)	50% (*n* = 2)	—	86% (*n* = 14)	65% (*n* = 20)	50% (*n* = 4)	70% (*n* = 40)
	Wood pigeon (*Columba palumbus*)	56% (*n* = 18)	100% (*n* = 4)	67% (*n* = 9)	47% (*n* = 15)	25% (*n* = 4)	56% (*n* = 50)

(b)					
Order	Species (Latin name)	2013	2014	2015	Total
Passerines	Blackbird (*Turdus merula*)	100% (*n* = 3)	0% (*n* = 2)	50% (*n* = 2)	57% (*n* = 7)
	Blackcap (*Sylvia atricapilla*)			100% (*n* = 1)	100% (*n* = 1)
	Blue tit (*Cyanistes caeruleus*)	50% (*n* = 2)		0% (*n* = 1)	33% (*n* = 3)
	Bullfinch (*Pyrrhula pyrrhula*)			0% (*n* = 1)	0% (*n* = 1)
	Chaffinch (*Fringilla coelebs*)	100% (*n* = 2)	33% (*n* = 3)	0% (*n* = 2)	43% (*n* = 7)
	Dunnock (*Prunella modularis*)	100% (*n* = 1)	50% (*n* = 2)	14% (*n* = 7)	30% (*n* = 10)
	Goldfinch (*Carduelis carduelis*)	100% (*n* = 1)	50% (*n* = 2)	0% (*n* = 1)	50% (*n* = 4)
	Greenfinch (*Chloris chloris*)	0% (*n* = 1)	30% (*n* = 10)		27% (*n* = 11)
	Great tit (*Parus major*)	33% (*n* = 6)		0% (*n* = 2)	25% (*n* = 8)
	House sparrow (*Passer domesticus*)		40% (*n* = 5)	0% (*n* = 3)	25% (*n* = 8)
	Jackdaw (*Corvus monedula*)	50% (*n* = 2)	0% (*n* = 1)		33% (*n* = 3)
	Jay (*Garrulus glandarius*)		25% (*n* = 4)		25% (*n* = 4)
	Long tailed tit (*Aegithalos caudatus*)			50% (*n* = 2)	50% (*n* = 2)
	Magpie (*Pica pica*)		100% (*n* = 2)		100% (*n* = 2)
	Pied wagtail (*Motacilla alba*)	100% (*n* = 1)			100% (*n* = 1)
	Robin (*Erithacus rubecula*)	100% (*n* = 3)			100% (*n* = 3)
	Rook (*Corvus frugilegus*)		0% (*n* = 1)		0% (*n* = 1)
	Common Starling (*Sturnus vulgaris*)	100% (*n* = 1)	0% (*n* = 5)		17% (*n* = 6)
	Common whitethroat		100% (*n* = 1)		100% (*n* = 1)
	Yellowhammer (*Emberiza citrinella*)		0% (*n* = 4)	75% (*n* = 4)	38% (*n* = 8)
Galliformes	Grey partridge (*Perdix perdix*)		0% (*n* = 2)		0% (*n* = 2)
	Common pheasant (*Phasianus colchicus*)		33% (*n* = 3)		33% (*n* = 3)
	Red legged partridge (*Alectoris rufa*)	100% (*n* = 1)	50% (*n* = 4)	0% (*n* = 2)	43% (*n* = 7)
Gruiformes	Common moorhen (*Gallinula chloropus*)		0% (*n* = 1)		0% (*n* = 1)

*Note:* Columbid samples from the UK in 2011 are those reported in Lennon et al. (2013) and those from Senegal and Burkina Faso are reported in Dunn et al. (2023). Birds from Burkina Faso were caught during the winter of 2012–2013 but results are collated into 1 year (2012). ‘—’ indicates no birds of this species were sampled during this year.

**TABLE 2:** Results of binomial GLMs determining factors associated with *T. gallinae* prevalence in columbid populations for each of the most prevalent strains separately, and overall.

	Type A^b^			Type C			GEO^a^			Tcl‐1			Overall		
	Dev.	df	*p*	Dev.	df	*p*	Dev.	df	*p*	Dev.	df	*p*	Dev.	df	*p*
Year	**34.84**	**4, 455**	**< 0.001**	6.49	4, 459	0.166	**15.41**	**4, 466**	**0.004**	**16.61**	**4, 459**	**0.002**	**10.33**	**4, 459**	**0.035**
Country	**46.03**	**3, 455**	**< 0.001**	**18.14**	**3, 458**	**< 0.001**	**78.00**	**3, 465**	**< 0.001**	**13.39**	**3, 458**	**0.004**	**32.21**	**3, 458**	**< 0.001**
Species^a^/Diet	**16.30**	**9, 455**	**0.002**	**40.06**	**9, 464**	**< 0.001**	**34.13**	**2, 464**	**< 0.001**	*14.79*	*9, 464*	*0.097*	**25.61**	**9, 464**	**0.002**

*Note:* Comparisons are between full model and model without the term. Significant differences at *p* < 0.05 in bold; marginal differences at 0.05 < *p* < 0.1 are italicised.

Abbreviation: Dev., Deviance.

^a^ The GEO model did not converge when Species was included, so Diet was used as a proxy in this model only.

^b^ The Type A model was underdispersed, so a quasibinomial error structure was used.

Thirteen strains were identified in columbids by sequencing of the ITS region (Figure 2); those in turtle doves (Thomas et al. 2022), from Senegal (Dunn et al. 2023), and from UK columbids in 2012 (Stockdale et al. 2015) have been described elsewhere so are not discussed further here, although they are included in subsequent statistical analyses. Type A, Type C, GEO and Tcl‐1 were the more commonly identified strains, and are analysed further here. We report a new strain, named WQR‐Env (GenBank accession number PX940052), isolated from two woodpigeons at a single site in Essex, UK in 2014. This strain has 99% similarity to strain WQR, previously isolated from Australasian columbids (Peters and Raidal, unpublished data). Further sub‐typing at the FeDH region identified Type A subtype A1 in three stock doves and one woodpigeon at two sites in Essex, UK in 2014 and 2015. Three Type C subtypes were also identified: subtype C11 in a collared dove found dead in Essex, UK, in 2014; subtype C4 in a woodpigeon from France, 8 woodpigeons from three sites in Essex and two stock doves from Essex, and subtype C6 was isolated from a collared dove from France.

**FIGURE 1:**
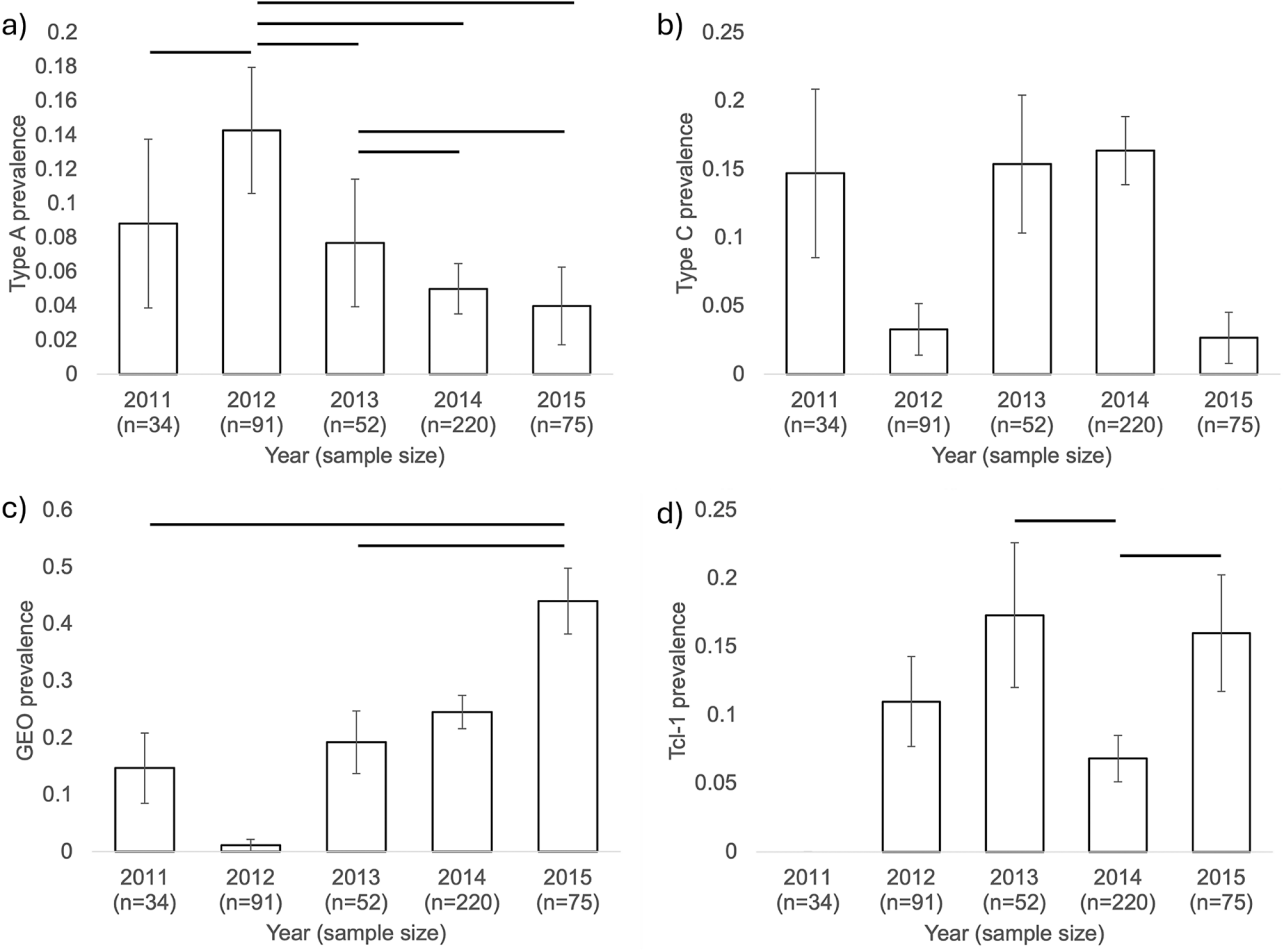
The prevalence in all columbids from Burkina Faso, France, Senegal and the UK of (a) Type A (b) Type C (c) GEO and (d) Tcl‐1 differed by Year (Table 2). Significant pairwise differences at *p* < 0.05 are denoted by lines above columns.

The prevalence of all common strains apart from Tcl‐1 and Type C also differed between years, countries and species (Table 2; Figure 1); the prevalence of GEO differed between species diets; the prevalence of Tcl‐1 only marginally differed between species, but differed between years and countries. The prevalence of Type A varied with year, being higher in 2012 than in all other years, and higher in 2013 than in 2014 and 2015 (Figure 1a). GEO prevalence was higher in 2015 than in both 2011 and 2013 (Figure 1c), and Tcl‐1 prevalence was lower in 2014 than in both 2013 and 2015 (Figure 1d).

Type A was more common in the UK (16.9% ± 2.9%) than in France (1.2% ± 1.2%) whereas GEO was more common in Senegal (52.3% ± 4.1%) than in both France (7.4% ± 2.9%) and the UK (7.2% ± 2.0%). Tcl‐1 had a higher prevalence in France (16.0% ± 4.1%) than in the UK (7.2% ± 2.0%). No pairwise differences were significant for Type C.

Type A prevalence was higher in both feral pigeons (25.0% ± 25.0%) and stock doves (19.0% ± 6.1%) than in woodpigeons (9.6% ± 4.1%). Type C prevalence was lower in stock doves (2.4% ± 2.4%) than in vinaceous doves (25.0% ± 25.0%), laughing doves (15.7% ± 4.0%), and collared doves (25.0% ± 11.1%), higher in collared doves than in turtle doves (12.4% ± 2.3%) and woodpigeons (7.7% ± 3.7%), and higher in laughing doves than in turtle doves. Overall prevalence was higher in collared doves (87.5% ± 8.5%) than in woodpigeons (57.7% ± 6.9%).

### 3.2 Prevalence in All UK Birds

Overall *T. gallinae* prevalence in all UK birds sampled was lower in 2015 (33.3% ± 7.6%) than in 2011 (85.7% ± 7.8%) and 2013 (81.6% ± 4.5%), and lower in 2014 (46.5% ± 5.0%) than in 2013 (Tables 1 and 3). Prevalence was lower in May (41.1% ± 6.0%) than in both June (74.2% ± 3.8%) and July (69.0% ± 8.7%), and was lower in fed sites (Fed: 58.4% ± 3.8%; Unfed: 69.2% ± 5.3%), although pairwise comparisons indicated that this effect was marginal (Table 3).

**TABLE 3:** Results of a binomial GLM determining factors associated with *T. gallinae* infection in UK passerines.

	Type A			Type C^a^			GEO^a^			Tcl‐1^a^			Overall		
	Dev.	df	*p*	Dev.	df	*p*	Dev.	df	*p*	Dev.	df	*p*	Dev.	df	*p*
Year	**47.32**	**4, 244**	**< 0.001**	**8.32**	**4, 245**	**0.032**	**15.45**	**4, 242**	**< 0.001**	**43.15**	**4, 245**	**< 0.001**	**29.21**	**4, 239**	**< 0.001**
Month	2.89	3, 247	0.408	5.65	3, 247	0.130	2.78	3, 247	0.391	**22.81**	**3, 244**	**< 0.001**	**20.97**	**3, 238**	**< 0.001**
County	1.93	1, 249	0.164	0.49	1, 249	0.484	1.23	1, 239	0.094	0.08	1, 249	0.775	1.01	1, 249	0.315
Site type	3.65	2, 248	0.162	1.70	2, 248	0.428	0.67	2, 240	0.466	1.20	2, 243	0.109	2.84	2, 237	0.242
Family	**8.73**	**2, 242**	**0.013**	3.14	2, 243	0.136	2.01	2, 240	0.102	0.01	2, 248	0.998	5.05	2, 237	0.080
Diet	7.08	3, 243	0.069	2.62	3, 244	0.344	**18.02**	**3, 241**	**< 0.001**	1.82	3, 247	0.618	3.11	1, 236	0.078
Fed	**8.40**	**1, 241**	**< 0.001**	1.70	1, 249	0.193	0.34	1, 249	0.563	1.34	1, 249	0.249	**16.41**	**3, 238**	**< 0.001**

*Note:* Statistics are from comparisons of the full model and the model with the term removed. Significant results are in bold; cells in grey indicate that this term was not included in the final model.

Abbreviation: Dev., Deviance.

^a^ The final model was underdispersed, so a quasibinomial error structure was used.

**FIGURE 2:**
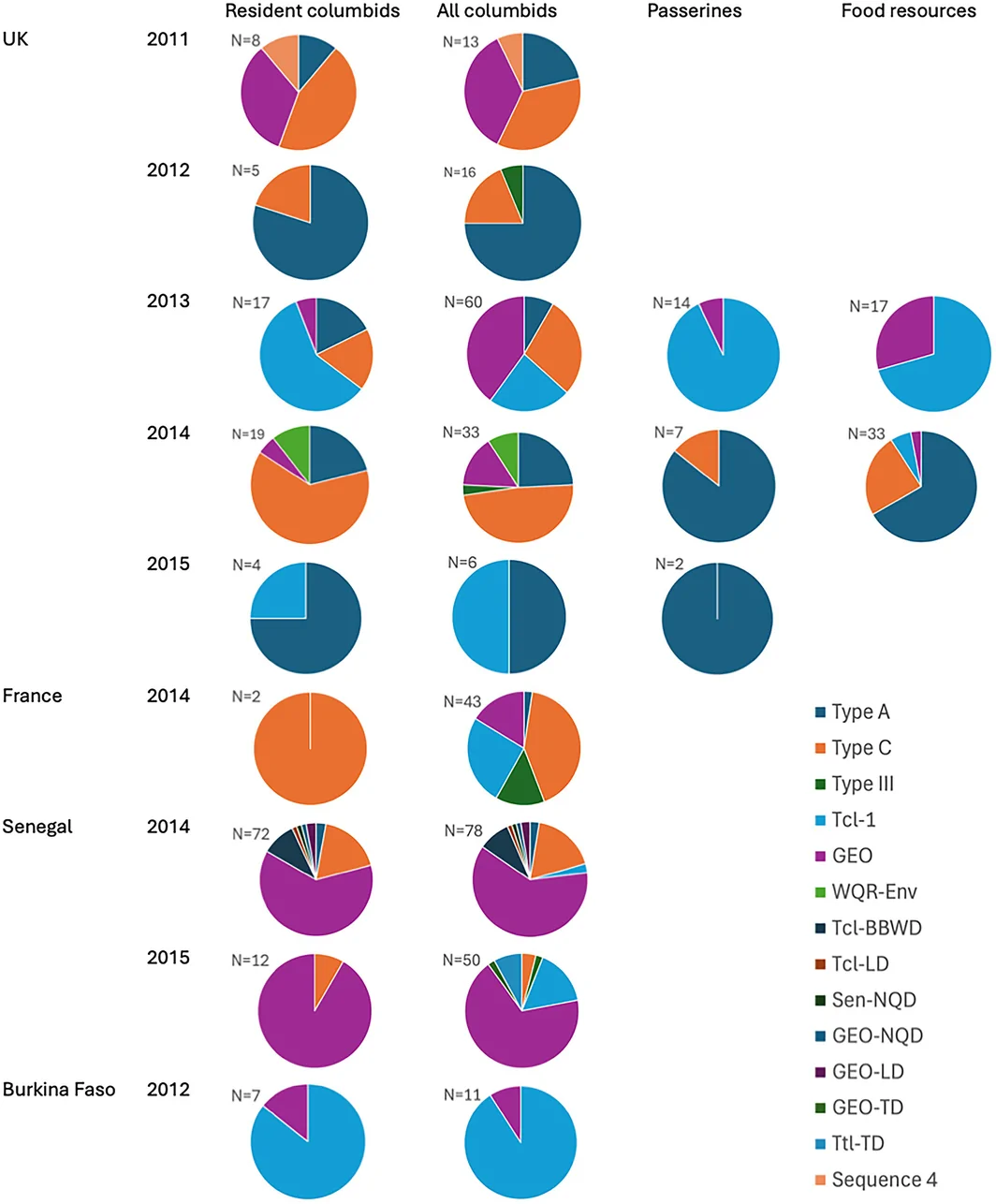
Pie charts reflecting the strain composition of *T. gallinae* infecting columbid populations, allowing the comparison of spatiotemporal variation in strain composition within resident columbid species only (excluding turtle doves) with all columbid species in different countries and between different years. Turtle dove data are from Lennon et al. (2013), Stockdale et al. (2015), and Thomas et al. (2022); data from Burkina Faso and Senegal are from Dunn et al. (2023). The strain composition infecting passerines and shared food resources is also shown for the UK during different years.

Strain diversity in non‐columbids was much lower than that found in columbids, with only the four most common strains identified (Figure 2), although it must be noted that sequencing success was relatively low (50% of positive samples returned good quality sequence). As in other studies (MacDonald et al. 2025), we also obtained inconclusive PCR results in a high proportion (46%) of passerine samples, which we omit from further analysis (see Appendix A for sample details). Type A was isolated from six species: one ring‐necked pheasant (*Phasianus colchicus*), one magpie (*Pica pica*), two yellowhammers (*Emberiza citrinella*), two greenfinches (Chloris chloris), one goldfinch (*Carduelis carduelis*), and one house sparrow (*Passer domesticus*). Sub‐typing at the FeDH region confirmed the presence of Type A subtype A1 in the yellowhammers, greenfinches and house sparrow. Type C was found only in one house sparrow, with sub‐typing confirming this to be subtype C4. GEO was found in one dunnock (*Prunella modularis*). Tcl‐1 was found in 8 individuals from 5 species: one red legged partridge (*Alectoris rufa*), two blackbirds (*Turdus merula*), two chaffinches (*Fringilla coelebs*), two robins (*Erithacus rubecula*), and one pied wagtail (*Motacilla alba*).

Type A prevalence was higher in 2012 (81.3% ± 10.1%) than all subsequent years, and lower in 2013 (5.3% ± 2.6%) than in both 2014 (14.1% ± 3.5%) and 2015 (12.8% ± 5.4%). Columbids (18.7% ± 3.2%) had a higher prevalence of Type A than passerines (7.9% ± 2.9%), and Type A prevalence was more than double at fed sites (17.3% ± 2.9%) than at unfed sites (7.7% ± 3.0%; Table 3), in stark contrast to overall positivity rates.

GEO was found only in 2 years, with a higher prevalence in 2013 (14.5% ± 4.1%) than 2014 (2.0% ± 1.4%), and a higher prevalence in granivorous species (20.4% ± 5.5%) than herbivorous species (2.1% ± 2.1%). Tcl‐1 was also only found in 2 years, being at higher prevalence in 2013 (22.4% ± 4.8%) than in 2015 (7.7% ± 4.3%). Prevalence was higher in June (14.4% ± 3.1%) than in July (3.4% ± 3.4%), with Tcl‐1 not detected in May or after July.

**FIGURE 3:**
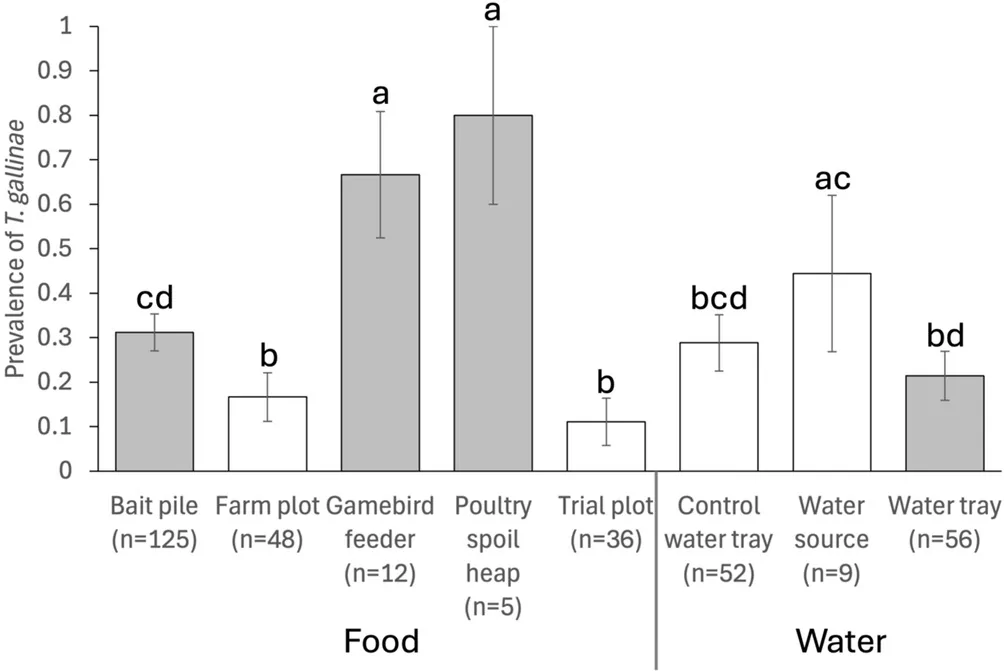
The prevalence of *T. gallinae* in different environmental resources. Bars show mean ±1 SE. Total *N* sampled includes repeated testing of the same resource over a season. Does not include samples for which the infection status was inconclusive. Shaded bars indicate anthropogenic resources; white bars are control or natural resources. Bars sharing a letter do not differ from one another at *p* < 0.05.

The results of an AMOVA reveal a borderline strong differentiation (FST value = 0.25 where > 0.25 is considered strong) (Table 4) (Hartl and Clark 1997) in the ITS strains infecting different columbid populations (defined according to year and country sampled), supporting the observed temporal and geographical variation displayed in Figure 3.

**TABLE 4:** AMOVA results.

Source of variation	df	Variance components	Percentage of variation	Fixation index FST
Among populations	7	0.1	24.88	0.25
Within populations	237	0.29	75.12	

*Note:* Populations = year + country. Groups = 1.

### 3.3 Percentage of Environmental Resources Testing Positive for *T. gallinae*

*T. gallinae* was detected in 27.4% of 343 samples collected from 51 food and water resources across 12 farmland sites over 2 years (Table 5; Figure 3), with 39% of resources (24 out of 62) from which multiple conclusive PCR results were obtained testing positive on more than one occasion (Table 5). The percentage of positive samples differed between resource types (GLM, Dev_7,331_ = 27.84, *p* < 0.001; Figure 3) and farms (GLM, Dev_11,335_ = 35.45, *p* < 0.001), with the highest positivity rates in gamebird feeders and the poultry spoil heap, and lowest positivity rates in trial plots and farm plots (Figure 3).

**TABLE 5:** Prevalence of *T. gallinae* infection detected in the food and water resources sampled during a season.

Year	Resource	Positive at least once during season	Positive on repeated samplings
2013	Food	59% (*n* = 17)	43% (*n* = 14)
	Water	80% (*n* = 5)	0 (*n* = 2)
2014	Food	73% (*n* = 22)	41% (*n* = 22)
	Water	70% (*n* = 20)	40% (*n* = 20)

*Note:* *N* indicates the total number of individual resources from which conclusive PCR results were obtained on at least one occasion, and the number of resources from which conclusive PCR results were obtained on multiple occasions.

*T. gallinae* was detected in 27.6% of samples collected from food resources. Positivity rates were higher during 2013 than 2014 (2013: 37.8% ± 5.4%; 2014: 21.7% ± 3.5%; Table 6), were higher in Essex than in East Anglia (Essex: 38.1% ± 4.0%; East Anglia: 7.7% ± 3.0%; Table 6), and were higher in May than in both June and July (May: 33.3% ± 8.7%; June: 24.7% ± 4.4%; July: 28.6% ± 4.6%; Table 6). High density feed sites had nearly three times the positivity rates of low density feed sites (high density: 36.9% ± %; low density: 13.3% ± %; Table 6), rainfall had a slight positive association with positivity rates of *T. gallinae* (Table 6), and increasing temperature had a small and marginally significant association with increased *T. gallinae* positivity (Table 6). Neither the amount of food resource remaining at the site nor the condition of the bait pile in terms of moisture were associated with *T. gallinae* positivity (Table 6).

**TABLE 6:** Results of binomial GLMMs determining factors associated with *T. gallinae* prevalence in food resources for each of the most prevalent strains separately, and overall.

	Water			Food (1)			Food (2)		
	χ^2^	df	*p*	χ^2^	df	*p*	χ^2^	df	*p*
Year	1.88	1	0.170	**5.99**	**1**	**0.014**	1.30	1	0.254
Month	**12.56**	**2**	**0.002**	**6.95**	**2**	**0.031**	**7.40**	**2**	**0.025**
County	1.00	1	0.316	**17.37**	**1**	**< 0.001**	**9.10**	**1**	**0.003**
Type	2.77	3	0.429	**10.01**	**1**	**0.002**	—	—	—
Rainfall	2.57	1	0.109	**6.08**	**1**	**0.014**	0.29	1	0.590
Temperature	**16.92**	**1**	**< 0.001**	*3.70*	*1*	*0.054*	**11.68**	**1**	**< 0.001**
Seed	—	—	—	—	—	—	1.59	2	0.451
State	**9.91**	**2**	**0.007**	—	—	—	0.262	2	0.877

*Note:* Comparisons are between the full model and model without the term. Significant differences at *p* < 0.05 in bold; marginal differences at 0.05 < *p* < 0.1 are italicised. Terms shaded in grey were non‐significant during univariate analysis, so were not included in the final model. Resource type within Farm were included as nested random effects in the water and Food (1) models to control for temporal replication of sampling across the same environmental resources; only Farm was included as a random term in the Food (2) model.

*T. gallinae* was cultured from 26.5% of 25 water resources sampled. Positivity rates were higher in May than in both June and July, and lower in June than in July (May: 58.3% ± 14.9%; June: 21.2% ± 5.1%; July: 25.6% ± 7.1%; Table 5). Mean temperature at resources positive for *T. gallinae* was higher than that at negative resources (Positive: 17.39°C ± 0.66°C; negative: 15.45°C ± 0.27°C).

### 3.4 Strain Identities of *T. gallinae* in Environmental Resources

In 2013, only two strains were detected from the shared resources: Tcl‐1 (*n* = 9) (Martínez‐Herrero et al. 2014) and GEO (*n* = 5) (Peters and Raidal, unpublished data). Only Tcl‐1 was detected in the water resources in 2013 (*n* = 3). In 2014, 10 food resources were infected with the Type A strain, 3 with Type C (Gerhold et al. 2008), one with Tcl‐1, and one was co‐infected with Type A and Type C. Eleven water resources were infected with Type A, four with Type C, one with Tcl‐1, one with GEO, and one was co‐infected with Type A and Type C.

## 4 Discussion

We found *T. gallinae* to be widely present in a range of environmental resources and host species. Any impact of *T. gallinae* infection on these hosts is unknown. This is the first record of sub‐clinical *T. gallinae* infection in many of the passerine species sampled, and we provide evidence of multiple strains circulating in this species group. *T. gallinae* prevalence differed between years for all strains within all host groups, and between countries for all strains in columbids, suggesting significant environmental effects on prevalence. Positivity rates in environmental resources were significant, with temperature linked to higher positivity rates in water and to a lesser degree in food. Overall, our data strongly suggest that food and water resources, especially those which are anthropogenic in origin, may be key in parasite transmission, particularly for the Type A strain (Becker et al. 2015). Changing the way that such resources are provided should offer an opportunity to limit parasite spread.

A high prevalence of *T. gallinae* infection persisted in the UK columbid population over a period of 4 years, and the parasite was more prevalent and diverse in columbids than passerines. This confirms that UK columbids are a key reservoir for *T. gallinae* infection (Amin et al. 2014; Lennon et al. 2013). Columbids worldwide tend to have a high prevalence of *Trichomonas*, although this varies by species, with 74% in a pan‐European study (Marx et al. 2017), 19%–62% across Mauritian columbid species (Bunbury et al. 2007), and 38%–79% across Australia and Papua New Guinea (Peters et al. 2020).

Although columbids appear to be a reservoir for *T. gallinae* on a wide geographical scale, here, the Type A strain is associated with the UK columbid population. Shared strains between countries could be the result of migrating birds. The GEO strain is most prevalent in Senegal, which is an over‐wintering site of turtle doves who migrate along the western Palaearctic flyway (Eraud et al. 2013; Requena et al. 2025). Turtle doves appear to be the source of the GEO strain in British columbid populations during 2013, which suggests that this strain may have spread between countries via this migration route. The spread of the Type A strain from Britain to Fennoscandia and subsequently to central Europe is thought to be via migrating chaffinches, although the ability of sub‐clinically infected birds to migrate long distances is unknown and warrants further investigation (Ganas et al. 2014; Lawson, Robinson, et al. 2011; Lehikoinen et al. 2013).

While the prevalence in passerines is higher than in many studies (e.g., 0% in Brazil and Slovenia (Zadravec et al. 2016; Martínez et al. 2023); 1% in Saudi Arabia (Albeshr and Alrefaei 2020)), 20% of common mynahs in Pakistan were found to carry the Type C C4 subtype (Farooq et al. 2018), and Quillfeldt et al. (2018) found a similar prevalence and strain diversity to our study in a wider range of bird species including passerines in Germany. The need to investigate other factors, such as host density, feeding practices and climate in order to understand the differences in the impact of trichomonosis outbreaks in different countries has already been highlighted (Lehikoinen et al. 2013), but the same also applies to the differences in sub‐clinical infection prevalence in passerines between different countries. The detection of the parasite in insectivorous species such as pied wagtails is particularly interesting as it suggests mechanisms of transmission that may be via the ingestion of contaminated insects or water sources.

Interestingly, the strain composition of *T. gallinae* infecting passerines reflects the dominant strains in the columbid population within the same year. This suggests that the *T. gallinae* strains are repeatedly spilling over from columbids to passerines in proportion to their frequency, so *T. gallinae* may not be able to independently persist in passerine populations without repeated exposure. Long‐term epidemiological studies in wild passerine populations, where the same individuals can be recaptured over the course of their lifetimes, would be invaluable in understanding the course of infection in passerines, as well as potential impacts of sub‐clinical infection on survival (as has been found in Pink Pigeons) (Bunbury, Jones, et al. 2008).

Birds sampled on sites with anthropogenic food provided, either deliberately or as a byproduct of farmland activities, were more than twice as likely to be infected by the Type A strain than those sampled on unfed sites. All passerines sampled were sub‐clinically infected, including those with the Type A strain. This finding is not unprecedented as dead passerines without macroscopic lesions have been found to be infected with the Type A strain (Ermgassen et al. 2016). These birds may have been sampled at the incubation stage of infection before clinical signs developed, or had the disease but only with microscopic lesions of necrotic ingluvitis (Ermgassen et al. 2016). Alternatively, they may be able to prevent the formation of clinical lesions by the parasite, supported by trials with columbids showing that infection with a low‐pathogenic strain provides immunity against subsequent infection with a virulent strain (Stabler 1948). If passerines can carry the Type A strain without showing clinical signs, this could result in further spread of the parasite and increased contact rates with other spillover hosts. While it remains a possibility that passerines may act as reservoir hosts and are now contributing to the maintenance of *T. gallinae* in the wild bird population, the lower strain diversity seen in passerines compared to columbids, and lack of any passerine‐specific *T. gallinae* strains, do suggest that passerines serve primarily as spillover hosts. Further work is required to determine whether T. *gallinae* would remain circulating within passerine populations without ongoing spillover, and the possibility remains that reducing spillover—likely through environmental resources—may reduce prevalence within passerine populations.

We provide the first evidence for the widespread presence of *T. gallinae* in a range of environmental resources, including both food and water, although our sample size is relatively modest for some resource types. Interestingly, the highest positivity rates were at anthropogenic sources either resulting from or targeting domestic or gamebirds. Unsurprisingly, parasites were found at high density feed sites nearly three times as often as at low density feed sites, suggesting that the careful management of feeding sites could be crucial in reducing parasite transmission among species using these resources (MacDonald et al. 2025). Temperature is crucial in the transmission of directly transmitted parasites across a range of host–parasite systems, influencing both parasite development and viability (Studer et al. 2010). We found temperature to influence the likelihood of parasite detection in both food and water resources, suggesting that increasing temperatures due to climate change may facilitate *T. gallinae* transmission in temperate environments, increasing the importance of early intervention to reduce environmental parasite presence.

The detection of *T. gallinae* in water sources containing high volumes of water, such as ponds, is surprising and could be the result of high re‐infection rates or longer persistence of the parasite in water with higher levels of organic matter (Purple et al. 2019; Purple and Gerhold 2015). It may be that edge habitats that are accessible to terrestrial birds may concentrate organic matter, increasing transmission likelihood. Interestingly, *T. gallinae* detection was more likely in natural water sources than in artificial water trays, with control water trays (which camera traps showed that birds—mostly pheasants—were still able to access) showing no difference in positivity to other water resources. This suggests that any treatment of artificial water resources for *T. gallinae* (either through antimicrobials or increased hygiene measures) may be ineffective.

The presence and persistence of *T. gallinae* in shared food resources is sufficient to be of conservation concern. *T. gallinae* was more likely to be detected in high density food sources such as seed piles which attract a high concentration of bird species foraging in a small area; other pathogens such as *Mycoplasma gallisepticum* are known to be transmitted via fomites at bird feeders (Dhondt et al. 2007; Moyers et al. 2018). *T. gallinae* was less likely to be detected in low density food sources with no supplementary food such as sown seed plots or uncropped cultivated margins that promote natural regeneration of vegetation from the seed bank, which encourage birds to forage at lower densities over a larger area. This suggests that if supplementary food is provided, it should be scattered over a wide area to encourage similar behaviour; indeed, a recent field trial demonstrated no increased likelihood of *T. gallinae* presence when seed was scattered at low densities (MacDonald et al. 2025).

This is especially important given our findings from strain composition comparisons between birds and environmental resources, as well as our finding of Type A presence being more than double in birds at fed sites compared to those at unfed sites. The strains detected from shared food resources in 2013 and 2014 are the only ones isolated from passerines in those years. In 2014, the Type A strain was recovered from the majority of food resources and it was the dominant strain found in passerines. The temporal variation in *T. gallinae* strain composition being mirrored in columbids, passerines and shared food resources, in addition to the lack of an association between strains and bird families, provides further evidence that *T. gallinae* infection is being shared between taxonomic families via an environmental transmission route, and suggests that shared food resources could be the main transmission route for the Type A strain.

Future work should focus on the drivers of temporal trends in *T. gallinae* strain composition in an effort to manage the occurrence and spread of the Type A strain, as well as better understand interactions between strains, both within and between individuals and species (Thomas et al. 2022). This can only be achieved by continuing to monitor *T. gallinae* infection in free‐ranging populations as part of longitudinal studies enabling variations in strain composition to be examined in relation to environmental and demographic factors. Our findings suggest that management of food resources is likely more feasible than that of water resources, and that farmland habitat providing food for birds at low densities and on a rotational basis (MacDonald et al. 2025) is likely to be the best way to reduce parasite transmission in the long‐term, given that the decline in many farmland bird populations has been linked to declining food supplies in both the breeding and non‐breeding seasons (Siriwardena et al. 2000, 2008), and *T. gallinae* is linked with mortality in greenfinches in both breeding and non‐breeding seasons (Robinson et al. 2010). More widely, for passerines and garden birds, given our findings of *T. gallinae* in food resources, further work investigating the role of bird feeders in pathogen and parasite transmission year‐round would be valuable to assess their potential role and how this could be managed (Dunn and Clegg 2025).

## Author Contributions

Rebecca C. Thomas, Jenny C. Dunn, Antony J. Morris and Simon J. Goodman designed the study, Rebecca C. Thomas and Jenny C. Dunn collected field data, Rebecca C. Thomas and Helen Hipperson contributed to laboratory analysis. Rebecca C. Thomas and Jenny C. Dunn carried out statistical analyses, and all authors contributed towards writing and editing the manuscript.

## Funding

This work was supported by the Royal Society for the Protection of Birds; Natural England; NERC Biomolecular Analysis Facility, NBAF873.

## Disclosure

Benefit‐Sharing Statement: A research collaboration was developed with scientists from France, with all collaborators offered coauthorship or included in the acknowledgements, depending on their level of input. The results of the research have been shared with the broader scientific community through appropriate public databases and data repositories (see Data Availability Statement). The research addresses a primary concern, in this case the transmission of the potentially lethal *Trichomonas gallinae* parasite in wild birds. Sample collection in the UK was carried out under licence from the Home Office, with bird ringing carried out under licence from the British Trust for Ornithology. Sample collection in western France was carried out thanks to study sites piloted by the French National Game and Wildlife Agency (ONCFS). Samples were imported to the UK under Defra Import Licences PATH/201/2012/1, PATH/201/2012/2 and PATH/15/0482.

## Conflicts of Interest

The authors declare no conflicts of interest.

## Data Availability

New *Trichomonas gallinae* sequence data are available on GenBank under Accession Number PX940052. R code is available for the environmental analysis at 10.6084/m9.figshare.33204864, and for the reservoir host analysis at 10.6084/m9.figshare.33204900. Datasets are available for the reservoir host analysis at 10.6084/m9.figshare.33204915 (columbid analysis) and 10.6084/m9.figshare.33204924 (UK bird analysis), and for the environmental analysis at 10.6084/m9.figshare.33204936 (food model 1), 10.6084/m9.figshare.33204945 (food model 2) and 10.6084/m9.figshare.33204957 (water model).

## Appendix A

Samples from (a) Birds and (b) Environmental resources, where PCR results were inconclusive for the presence of *T. gallinae*, with a smear on the gel suggesting the presence of poor quality DNA, split by year, for the samples collected during this study. The number in brackets is the total number of samples collected in each category per year, including inconclusive samples. ‘—’ indicates no samples were inconclusive for this sample type in this year.

**Table:**

	2013	2014	2015
**(a)**			
Blackbird	—	—	1 (3)
Blackcap	—	—	4 (5)
Blue tit	7 (9)	—	4 (5)
Bullfinch	—	—	1 (2)
Chaffinch	2 (4)	—	12 (14)
Chiffchaff	—	—	1 (1)
Collared dove	—	—	1 (1)
Dunnock	5 (6)	—	5 (12)
Goldfinch	1 (2)	—	13 (14)
Greenfinch	1 (2)	2 (12)	7 (7)
Great tit	7 (13)	—	7 (9)
House sparrow	—	1 (6)	7 (10)
Jackdaw	—	1 (2)	—
Moorhen	1 (1)	—	—
Pheasant	—	—	1 (1)
Red legged partridge	—	—	1 (3)
Robin	—	—	1 (1)
Rook	—	—	1 (1)
Starling	—	1 (6)	—
Stock dove	—	2 (22)	2 (6)
Whitethroat	—	1 (2)	1 (1)
Woodpigeon	1 (10)	—	6 (11)
**(b)**			
Bait pile	2 (48)	6 (77)	
Farm plot	1 (7)	2 (41)	
Trial plot	—	4 (26)	
Gamebird feeder	1 (12)	—	
Water (natural)	21 (9)	—	
Water tray	—	9 (56)	
Control water tray	—	8 (52)	
